# A Case of Type 2 Autoimmune Pancreatitis Suggesting Two Potential Disease Activity Markers

**DOI:** 10.1016/j.gastha.2025.100635

**Published:** 2025-02-01

**Authors:** Kazuki Natsui, Seiichi Yoshikawa, Ayano Kagata, Yusuke Horibata, Hiroyuki Usuda, Manabu Takeuchi, Shuji Terai

**Affiliations:** 1 Department of Gastroenterology, Nagaoka Red Cross Hospital, Nagaoka, Niigata, Japan; 2 Division of Gastroenterology and Hepatology, Graduate School of Medical and Dental Sciences, Niigata University, Niigata, Japan; 3 Department of Pathology, Nagaoka Red Cross Hospital, Nagaoka, Niigata, Japan

**Keywords:** AIP, Type 2 AIP, GEL, IL-8, LRG

## Abstract

Autoimmune pancreatitis (AIP) is divided into 2 main subtypes: type 1 AIP (AIP-1) and type 2 AIP (AIP-2). This report describes a young woman diagnosed with AIP-2 using endoscopic ultrasound–guided tissue acquisition. AIP-2 has been reported less frequently than AIP-1 as serologic abnormalities and other organ involvement except for inflammatory bowel disease are absent. Additional laboratory analyses suggest that serum interleukin-8 and leucine-rich alpha-2 glycoprotein are more efficient disease activity biomarkers than conventional C-reactive proteins. We hope that this case report will contribute to the discovery of diagnostic biomarkers for AIP-2.

## Introduction

Autoimmune pancreatitis (AIP) has 2 subtypes: type 1 AIP (AIP-1), which is a pancreatic manifestation of immunoglobulin G4 (IgG4)–related disease, and type 2 AIP (AIP-2), characterized by granulocytic epithelial lesions (GELs) and association with inflammatory bowel disease, especially ulcerative colitis (UC).^1^ AIP-2 is less frequently reported than AIP-1 due to the absence of serological biomarkers like IgG4.^2^ This report describes a patient with AIP-2 and interesting trends in 2 serum markers, interleukin (IL)-8 and leucine-rich alpha-2 glycoprotein (LRG), along with a literature review.

## Case Report

**Figure 1:**
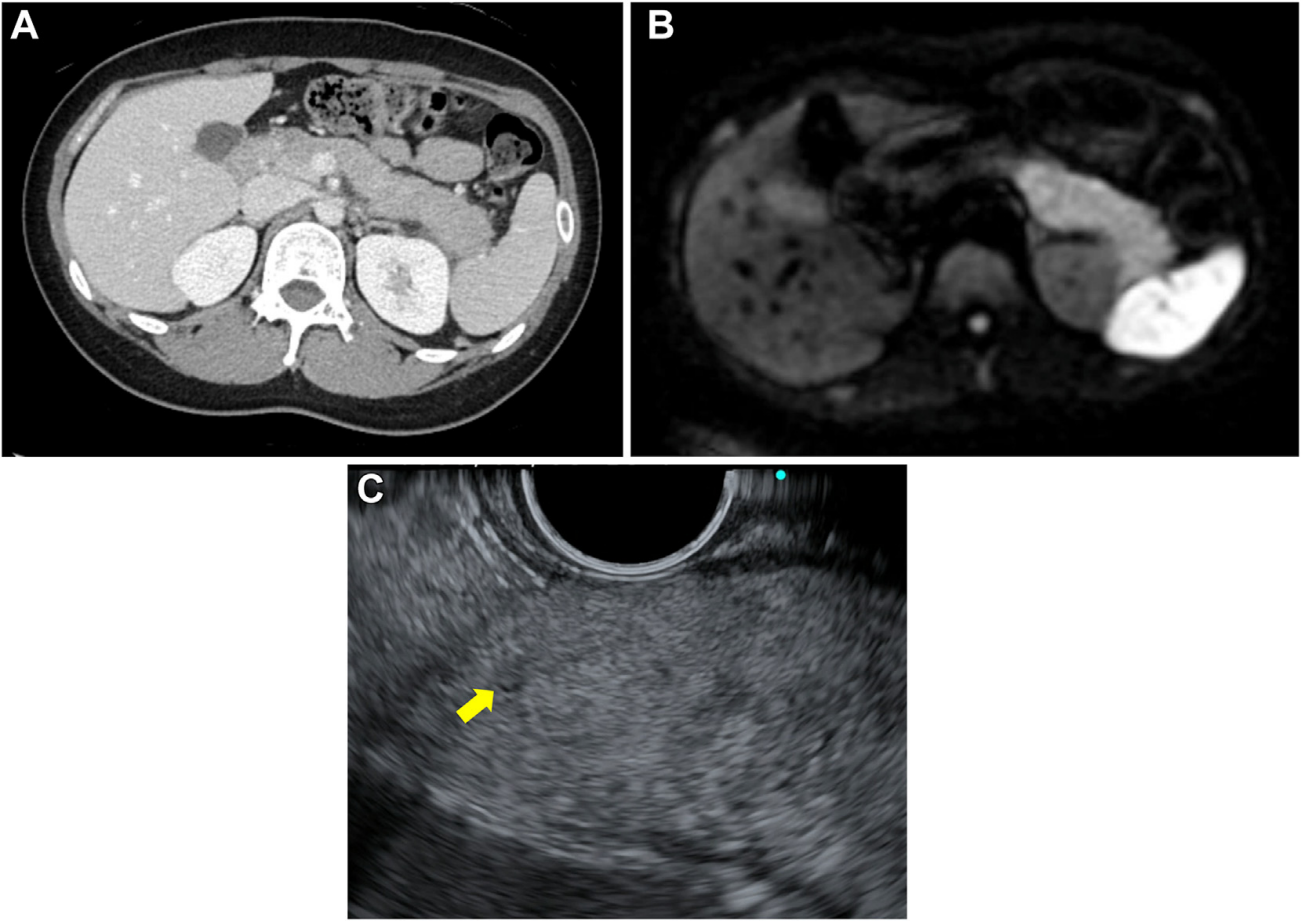
Various images with findings of the pancreatic body and tail at the initial visit. (A) Contrast-enhanced computed tomography shows swelling, with heterogeneous poor contrast. (B) DWI shows a high-intensity area. (C) EUS shows slight swelling and high-echo parenchyma. Yellow arrow shows an edematous MPD.

A 28-year-old woman without a history of drinking presented with abdominal pain in her epigastric region and left upper abdomen that had been persisting for the last month (day 1). Laboratory test showed a slightly elevated inflammatory reaction (white blood cells 6720/μL, reference range: 3300–8600/μL; and C-reactive protein 0.76 mg/dL, reference range: <0.014 mg/dL) and elevated pancreatic amylase (491 U/L, reference range: 16–52 U/L). The patient’s IgG4 level was 9.6 mg/dL (within the normal range). Contrast-enhanced computed tomography revealed swelling in the pancreatic body and tail, with heterogeneous poor contrast and increased peripancreatic adipose tissue concentration (Figure 1A). Diffusion-weighted imaging (DWI) revealed a high-intensity area in the pancreatic body and tail (Figure 1B).

**Figure 2:**
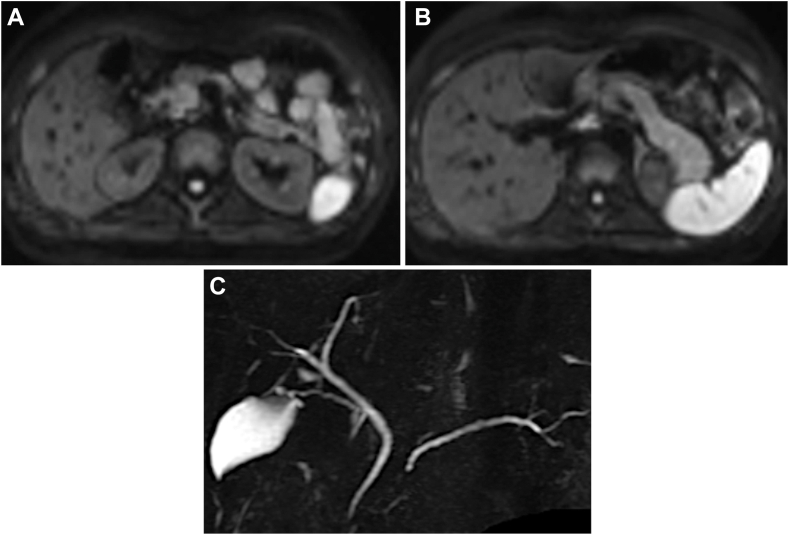
Magnetic resonance imaging images at the second admission. (A and B) DWI shows high-intensity areas in the pancreatic head (A) in addition to the body and tail (B). (C) Magnetic resonance cholangiopancreatography shows a narrowing of the MPD of the head and tail and slight enlargement of the body duct.

The patient was diagnosed with acute pancreatitis and admitted to our hospital, and despite conservative treatment, the pancreatitis symptoms persisted. Although the serum IgG4 levels were normal, endoscopic ultrasonography (EUS) and endoscopic retrograde cholangiopancreatography were performed, considering the possibility of AIP-1, gallstone pancreatitis, and pancreaticobiliary maljunction. EUS showed slight swelling and a high-echo parenchyma of the pancreatic body and tail, although AIP-1 was not strongly suggestive, and there were no findings of gallstones or pancreaticobiliary maljunction (Figure 1C). The patient recovered temporarily and was discharged on day 23, but was admitted on day 45 with a recurrence of epigastric pain. DWI revealed a high-intensity area in the pancreatic head beside the body and tail (Figure 2A and B), and magnetic resonance cholangiopancreatography revealed narrowing of the main pancreatic duct (MPD) of the head and tail and slight enlargement of the body duct (Figure 2C). EUS showed low-echo parenchyma with spotty high-echo also in the head. EUS–guided tissue acquisition (EUS-TA) was performed on the swollen pancreatic tail. However, the pathological findings revealed only pancreatic acinar tissue with mild inflammation.

**Figure 3:**
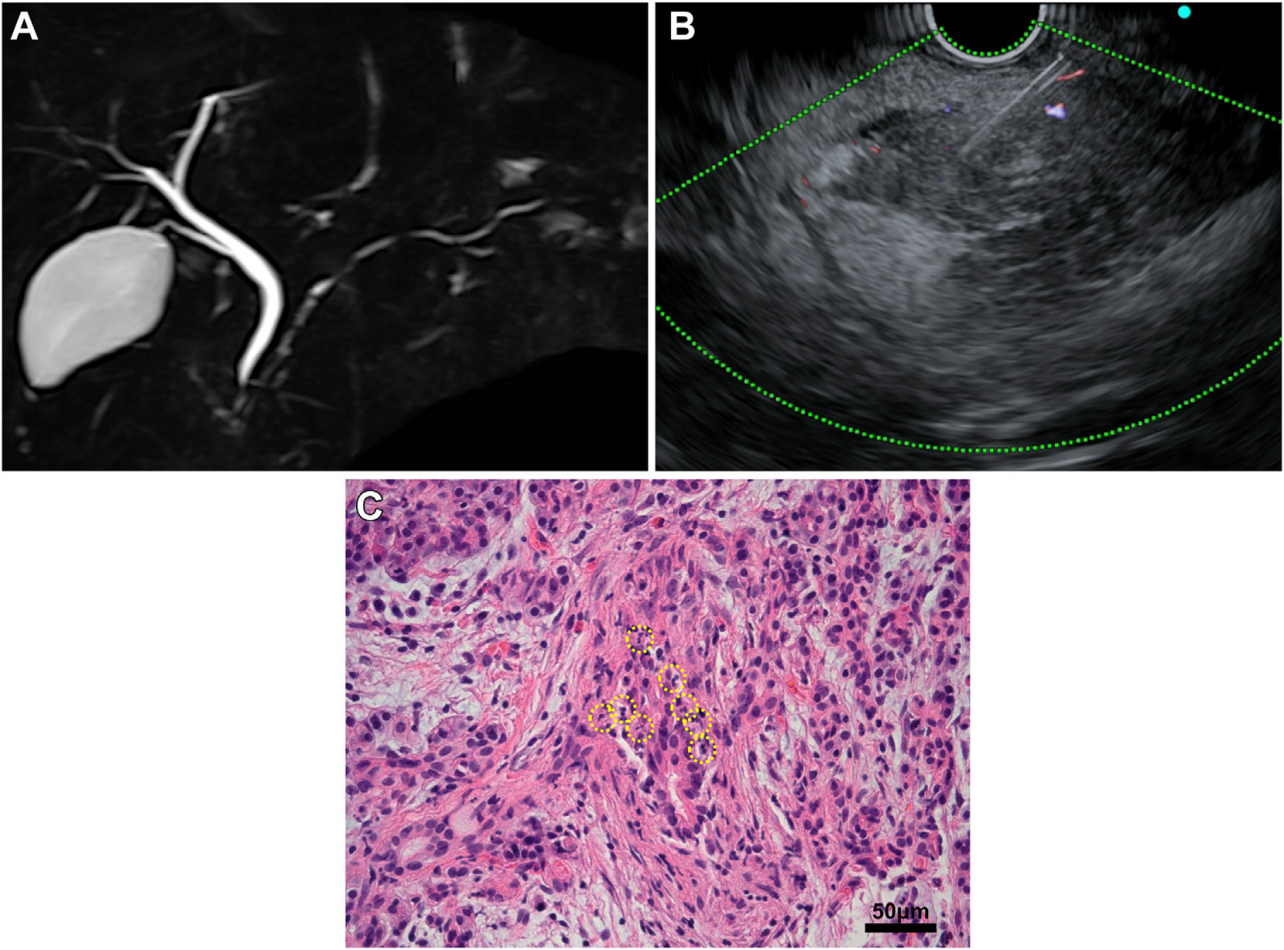
Various images at symptom exacerbation. (A) Magnetic resonance cholangiopancreatography shows the entire narrowing of the MPD. (B) Second EUS-guided tissue acquisition is performed on the pancreatic head. (C) Pathological findings detects GELs. Yellow circles show neutrophils.

**Figure 4:**
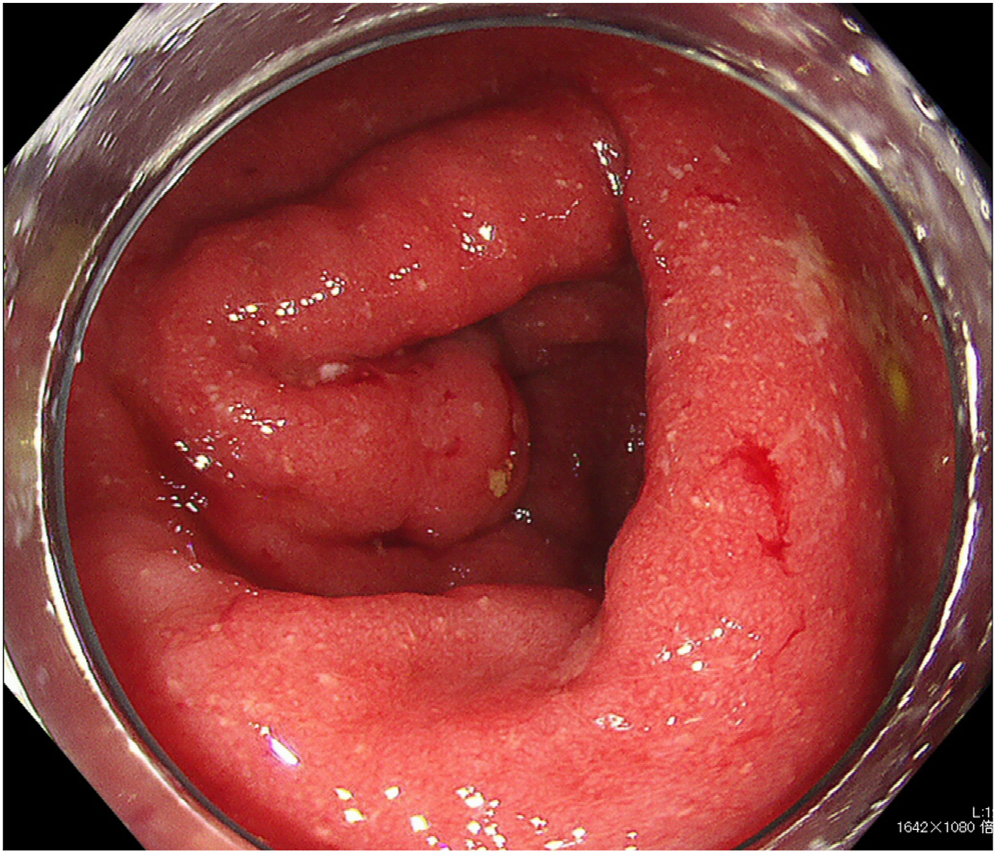
Colonoscopy image. Reddish edematous mucosa with pyogenic deposits only seen in the ascending colon.

**Figure 5:**
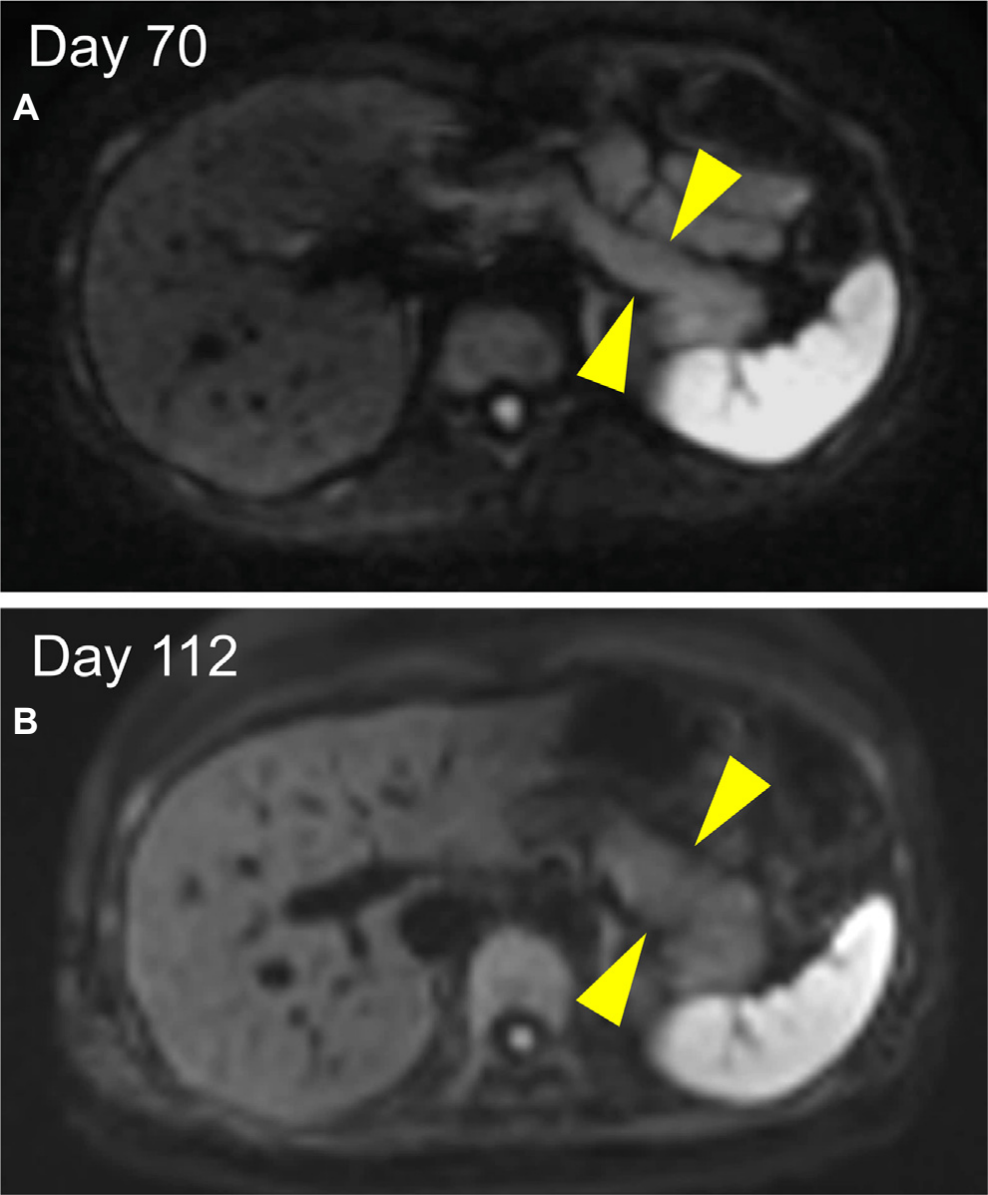
DWI images after treatment introduction. (A and B) DWI images on day 70 and 112. Yellow arrowheads show the pancreatic body and tail with high intensity area.

Subsequently, a high-intensity area of DWI spread and narrowing of the MPD were observed throughout the pancreas (Figure 3A). A second EUS-TA on the pancreatic head detected pathological findings of pancreatic duct destruction with neutrophilic infiltration in and around the small pancreatic lobes, that is, GEL, which met the level 1 histological criteria of the International Consensus Diagnostic Criteria (Figure 3B and C).^1^ There were no IgG4-positive plasma cells. Colonoscopy revealed a small UC area in the ascending colon, which was pathologically consistent. (Figure 4). Based on these findings, the patient was diagnosed with AIP-2 and was administered oral prednisolone and 5-aminosalicylic acid. Abdominal symptoms and imaging findings, including a swollen pancreas and narrowing of the MPD, were relieved, and the patient was discharged on day 70. During outpatient observation, mild abdominal pain was noted; however, the steroids were tapered off without dose escalation (Figure 5). To date, no instances of relapse have been reported.

**Figure 6:**
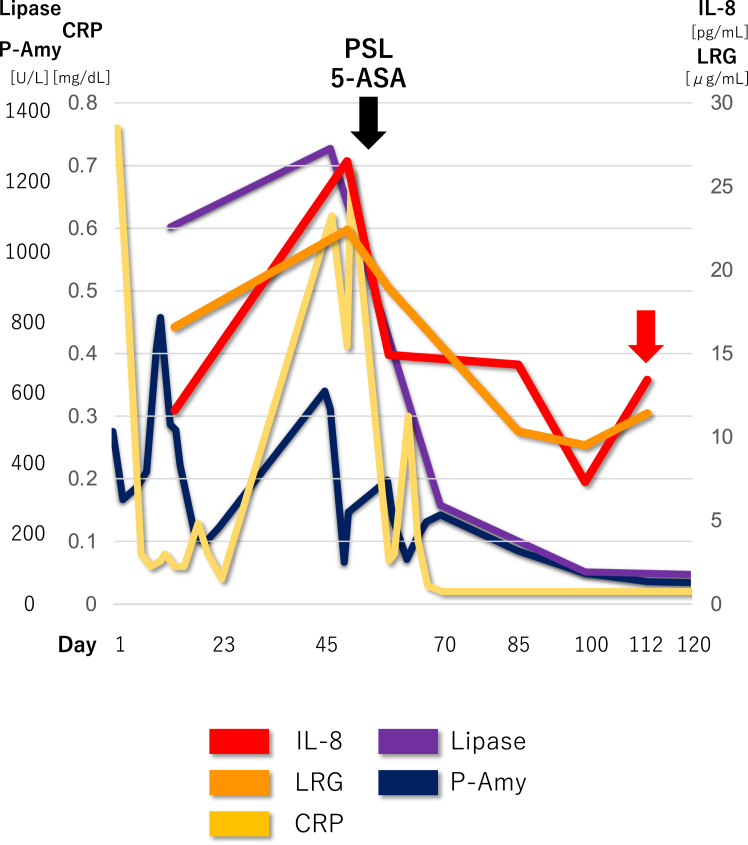
IL-8 and LRG trends with other serological indicators during the clinical course. Line graphs of IL-8 (pg/mL), LRG (μg/mL), C-reactive protein (mg/dL), pancreatic amylase (U/L), and pancreatic lipase (U/L).

Later analyses of IL-8 and LRG using preserved sera to search for candidate AIP-2 biomarkers were performed and the results are shown in Figure 6, together with other serological indicators.

## Discussion

AIP is a distinct form of pancreatitis, clinically characterized by obstructive jaundice with or without a pancreatic mass, histologically by lymphoplasmacytic infiltrate and fibrosis, and by a dramatic responds to steroids.^1^ There are 2 main forms: AIP-1 and AIP-2. AIP-1 is common in men in their 60s with high-level serum IgG4 levels, likely as a pancreatic manifestation of IgG4–related disease. In 75% of patients, AIP-1 is caused by obstructive jaundice due to pancreatic head swelling or IgG4-related sclerosing cholangitis, although approximately one-third of patients present with abdominal pain at onset.^3^^,^^4^

AIP-2, first proposed by Notohara et al. and Zamboni et al.,^5^^,^^6^ is more common in young men in their 40s.^4^^,^^7^^,^^8^ Compared to AIP-1, epigastric pain and acute pancreatitis occur more frequently in AIP-2, affecting >50% of patients.^4^^,^^8^ Approximately 30% of patients with AIP-2 have conditions associated with inflammatory bowel disease, especially UC.^9^ However, AIP-2 lacks serologic abnormalities and other organ involvement seen in AIP-1, making definitive diagnosis challenging without histology. This may explain why type 2 AIP is less frequently reported.^2^^,^^9^ Steroid treatment is effective for both forms, but relapse rates are approximately 60% in AIP-1 during tapering and <10% in AIP-2.^3^^,^^7^^,^^8^^,^^10^

Histologically, AIP-2 is characterized by idiopathic duct-centric pancreatitis with GELs, where neutrophils are prominent in the pancreatic ducts, and duct epithelium destruction is common.^1^^,^^5^ The pathogenesis of AIP-2 is not fully understood, but the infiltration of Th17 cells around the ducts and their release of IL-17, IL-21, IL-22, and IL-23 may play a role.^11^ IL-8 is overexpressed in the ductal or ductular epithelium and CD3^+^ T cells in AIP-2, stimulated by IL-17, which promotes GEL development.^12^ LRG, a disease activity biomarker in UC, is influenced by IL-6, tumor necrosis factor alpha, and IL-22.^13^^,^^14^

In Figure 6, both IL-8 and LRG present similar trends, which were consistent with the course of pancreatitis, including the degree of abdominal pain. Interestingly, the patient experienced mild abdominal pain during outpatient observation (Figure 6, red arrow), while C-reactive protein levels were within the normal range, IL-8 and LRG levels were elevated, and DWI showed exacerbated swelling in the pancreatic body and tail (Figure 5B). After the initial admission, the patient’s abdominal pain was predominantly in the left upper quadrant, consistent with the pancreatitis lesion, and there were no UC symptoms, such as diarrhea or bloody stools. These findings suggest that the IL-8 and LRG trends reflect AIP-2 disease activity. To confirm the validity of serum IL-8 and LRG as biomarkers for AIP-2, further evaluation with a larger number of diverse patients. We hope that this case report will play a role facilitating the discovery of AIP-2 diagnostic biomarkers.
